# Breastfeeding and employed mothers in Ethiopia: legal protection, arrangement, and support

**DOI:** 10.1186/s13006-021-00392-2

**Published:** 2021-06-14

**Authors:** Ermiyas Mulu Kebede, Benyam Seifu

**Affiliations:** College of Medicine and Health Science Ambo University, Ambo, Ethiopia

**Keywords:** Breastfeeding, Employed mothers, Law, Ethiopia

## Abstract

**Background:**

Breastfeeding is the single, most cost-effective intervention to reduce worldwide child mortality. Women empowerment interventions have positive impacts on child and maternal nutritional, and health status. Women’s employment and economic participation in Ethiopia have shown progress over the past three decades. However, consistent evidence indicated that maternal employment is often negatively associated with optimal breastfeeding in Ethiopia. The existence and enforcement of breastfeeding law, arrangement, and support in the workplace have vital roles in protecting employed mothers’ ability and right to breastfeed upon return to work from maternity leave. This commentary compared the breastfeeding laws, policies, and arrangements in Ethiopia with international standards, recommendations, and evidence-based practices.

**Workplace breastfeeding policies in Ethiopia:**

Public legislations of Ethiopia poorly protect the breastfeeding right of most new mothers. Ethiopian revised Labor Proclamation (No.1156/2019) incorporates most of the International Labour Organization maternity protection recommendations. However, it poorly safeguards breastfeeding rights and abilities of employed women. The provided maternity leave period is also shorter than the recommended exclusive breastfeeding duration. The revised Federal Civil Servant Proclamation of Ethiopia (NO.1064/2017) mandates the establishment of a nursery in government institutions where female civil servants could breastfeed and take care of their babies in a private room. Though, it protects only a small proportion of working mothers in Ethiopia, as majority women employed in the agriculture and informal economy sectors. So far, there are no notable workplace breastfeeding arrangements and support for employed mothers by employers and other initiatives. The ILO recommendation and experience of other middle income and low-income countries can be legal and practical grounds for establishment of breastfeeding-friendly workplace in Ethiopia.

**Conclusions:**

The lack of workplace breastfeeding laws, arrangements, and supports in Ethiopia limits mothers’ right to practice optimal breastfeeding. Policymakers, the government, and all concerned bodies should give due attention to enacting and enforcing sound laws and arrangements that will enable employed mothers to practice optimal breastfeeding upon return to work.

## Background

Optimal breastfeeding is the single most cost-effective intervention to reduce child mortality in developed and developing countries [1]. The impacts of breastfeeding on child mortality and morbidity, short-term and long-term child and maternal health, economic gain, and influence on future human capital development are well documented facts [2, 3]. On this basis, World Health Assembly Member States in 2002 established exclusive breastfeeding and continued breastfeeding up to 2 years as the norm and the natural way to feed infants and young children [4].

Women’s employment is one of the means for achieving the global effort of gender equality. Women empowerment interventions have positive impacts on child and maternal nutritional and health status [5]. Despite this fact, the changing composition of the workforce, especially women’s increased participation rates, has made it a great challenge to find ways for breastfeeding to be compatible with work [6, 7]. The International Labour Organization (ILO) indicated, returning to paid work is the main reason for the poor practice of breastfeeding among working mothers [8].

Suboptimal breastfeeding is among the main factors contributing to the high burden of child mortality, morbidity, and malnutrition in Ethiopia [9, 10]. Consistent evidence from diverse parts of Ethiopia indicated that maternal employment is often positively associated with suboptimal breastfeeding. Higher prevalence of nonexclusive breastfeeding, delayed breastfeeding initiation, and early cessation of breastfeeding were recorded among employed mothers compared to unemployed counterparts [11–15]. The progressing trend of women’s employment and economic participation in Ethiopia entail adequate legal protection, arrangement, and support for breastfeeding in the work place [16]. This commentary compared the breastfeeding laws, policies, and arrangements in Ethiopia with international standards, recommendations, and evidence-based practices.

## Legal protection of breastfeeding in the workplace

Different international, regional, and national laws protect motherhood and childhood worldwide. Enabling mothers to continue breastfeeding upon return to work is one of the essential elements of maternity protection. As early as 1919, the ILO acknowledged that breastfeeding was an integral part of motherhood and reproduction and deserved protection at the workplace [17]. The ILO Convention No. 183 emphasizes that a working mother should be entitled to one or more daily breaks or a daily reduction of hours of work to breastfeed her child.

*A woman shall be provided with the right to one or more daily breaks or a daily reduction of hours of work to breastfeed her child. (Convention No. 183, Article 10 (1). The period during which nursing breaks or the reduction of daily hours of work are allowed, their number, the duration of nursing breaks, and the procedures for the reduction of daily hours of work shall be determined by national law and practice. These breaks or the reduction of daily hours of work shall be counted as working time and remunerated accordingly. (Convention No. 183, Article 10 (2)* [18]*.*

Breastfeeding law, policies, and arrangements at the workplace should ensure optimal breastfeeding practice, work productivity, and women’s right to equal work opportunity. Evidence shows that workplace breastfeeding arrangement and support for working mothers increase women’s work motivation, attendance, satisfaction, and productivity and is essential to meet Infant and young child feeding (IYCF) standards of breastfeeding [4, 19].

## Importance of breastfeeding-friendly workplace policies in Ethiopia

The breastfeeding right of most new mothers in Ethiopia is unprotected by public legislation and policies. The revised Labor Proclamation (No.1156/2019) incorporates most of the International Labour Organization maternity protection recommendation. However, it does not have specific legislation on breastfeeding protection and support in the workplace [20]. The revised Federal Civil Servant Proclamation of Ethiopia (No.1064/2017) enacts the establishment of a nursery in government institutions, where female civil servants could breastfeed and take care of their babies [21]. The Civil Servant Proclamation protects only a small proportion of working mothers in Ethiopia, as majority of women are employed in other sectors. Besides, we found poor implementation and enforcement of breastfeeding act under Proclamation No.1064/2017. As far as we witnessed, most of the government institutions do not have nursery facilities, and workplace breastfeeding arrangements and support for employed mothers by employers and other initiatives are nonexistent.

Maternity leave is one of the opportunities that will promote breastfeeding for working mothers [8]. The duration of maternity leave provided under Labor Proclamation (No.1156/2019) meets ILO’s recommendation. However, the act has a narrow scope of maternity protection, as it does not cover most Ethiopian women employed in unpaid or underpaid agriculture sector and informal economy [22]. Unless the right to breastfeed is protected by additional act/s, the leave duration will not enable employed mothers to practice exclusive breastfeeding for 6 months and continue up to 2 years, as recommended by international and national guidelines.

The ILO recommendation and experience of other middle- and low-income countries can be legal and practical grounds for establishment of breastfeeding-friendly workplace in Ethiopia [23]. For instance, Philippines enacted a national policy to encourage, protect and support the practice of breastfeeding in the workplace. The “Expanded Breastfeeding Promotion Act of 2009” of Philippines mandates all private enterprises and government agencies to support breastfeeding through workplace breastfeeding policy, provision of space and time for breastfeeding, provision of breastfeeding information in the workplace [24].

All employed women in Ethiopia have a right to practice optimal breastfeeding and deserve laws, arrangements, and support in the workplace. The law and policy options could include; adopting ILO breastfeeding conventions, introducing inclusive and explicit laws that protect all employed mothers’ breastfeeding rights, and ensure implementation of the Federal Civil Servant Proclamation act. Besides, the involvement, mutual understanding, and coordination of all stakeholders are essential for establishing organizational, structural, and social arrangements of breastfeeding in the workplace. These emotional and physical arrangements could be made through initiatives like workplace awareness creation and promotion of breastfeeding, employer and colleague support of breastfeeding, the establishment of breastfeeding facilities and childcare centers, and flexible work arrangement etc. [25]. Although it might entail a detailed economic and cost-benefit analysis, extending the current maternity leave duration to 6 months might help exclusive breastfeeding.

## Conclusions

The absence of breastfeeding laws, arrangements, and supports for employed mothers limits their ability and right to practice optimal breastfeeding. Policymakers, governments, and all concerned bodies should give due attention to enacting and enforcing sound laws and establishing arrangements and supports that will enable employed mothers to practice optimal breastfeeding upon return to work. Future researchers could focus on assessing the consequences of the problem and contextualizing and piloting different breastfeeding-friendly workplace interventions.

## Data Availability

Not applicable.

## Abbreviations

CSA: Central Statistical Agency
EBF: Exclusive Breastfeeding
EDHS: Ethiopia Demography and Health Survey
FDRE: Federal Democratic Republic of Ethiopia
ILO: International Labour Organization/Office
IYCF: Infant and young child feeding
